# Determination of the Main Ergot Alkaloids and Their Epimers in Oat-Based Functional Foods by Ultra-High Performance Liquid Chromatography Tandem Mass Spectrometry

**DOI:** 10.3390/molecules26123717

**Published:** 2021-06-18

**Authors:** Laura Carbonell-Rozas, Laura Gámiz-Gracia, Francisco J. Lara, Ana M. García-Campaña

**Affiliations:** Department of Analytical Chemistry, Faculty of Sciences, University of Granada, Avda. Fuente Nueva s/n, 18071 Granada, Spain; rozas@ugr.es (L.C.-R.); lgamiz@ugr.es (L.G.-G.); frjlara@ugr.es (F.J.L.)

**Keywords:** ergot alkaloids, oat, food supplements, QuEChERS, UHPLC–MS/MS

## Abstract

An ultra-high performance liquid chromatography coupled to tandem mass spectrometry method is proposed for the determination of the major ergot alkaloids (ergometrine, ergosine, ergotamine, ergocornine, ergokryptine, ergocristine) and their epimers (ergometrinine, ergosinine, ergotaminine, ergocorninine, ergokryptinine, and ergocristinine) in oat-based foods and food supplements. A modified QuEChERS (quick, easy, cheap, effective, rugged, and safe) procedure was applied as sample treatment, reducing the consumption of organic solvent and increasing sensitivity. This method involved an extraction with acetonitrile and ammonium carbonate (85:15, *v/v*) and a clean-up step based on dispersive solid-phase extraction, employing a mixture of C18/Z-Sep+ as sorbents. Procedural calibration curves were established and limits of quantification were below 3.2 μg/kg for the studied compounds. Repeatability and intermediate precision (expressed as RSD%) were lower than 6.3% and 15%, respectively, with recoveries ranging between 89.7% and 109%. The method was applied to oat-based products (bran, flakes, flour, grass, hydroalcoholic extracts, juices, and tablets), finding a positive sample of oat bran contaminated with ergometrine, ergosine, ergometrinine, and ergosinine (total content of 10.7 μg/kg).

## 1. Introduction

Ergot alkaloids (EAs) are mycotoxins produced mainly by fungi of the Claviceps genus, as Claviceps purpurea, which parasitize the seeds of living plants (such as rye, triticale, wheat, oat, and barley) and replace the grain with fungal structures known as sclerotia that contain alkaloid substances. The sclerotia are harvested together with the cereals or grass and, although improvements in agricultural practices have significantly reduced their presence, EAs can still be found in cereal-based foods and feed. The ingestion of these contaminated products might cause intoxications in humans and animals, and illnesses, such as ergotism, characterized by symptoms, such as abdominal pain, vomiting, burning sensation of the skin, insomnia, and hallucinations [1,2,3]. For that reason, the European Commission (EU) has established a maximum level of 0.5 g/kg for ergot sclerotia in most unprocessed cereals [4]. Although regulatory limits for EAs have not been established yet, they will probably be included in the near future in mycotoxin legislation [5]. Moreover, the European Food Safety Authority (EFSA) has suggested a tolerable daily intake for total EAs of 0.6 μg/kg of body weight/day and an acute reference dose of 1 μg/kg body weight [6].

More than 40 different EAs are known to date, whose production depends on several factors, such as the maturity of the sclerotia, the fungal strain, the host cereal, the geographical region, and the climate conditions. For these reasons, EFSA has stated that a wide collection of analytical results is required in order to define the variability of EAs in food and feed commodities, paying special attention to processed foods [7]. The most predominant EAs, which are already included in European recommendations, are ergometrine (Em), ergotamine (Et), ergosine (Es), ergocristine (Ecr), ergokryptine (Ekr), and ergocornine (Eco). These analytes undergo epimerization with respect of its center of symmetry of C8, being the corresponding epimers: ergometrinine (Emn), ergotaminine (Etn), ergosinine (Esn), ergocristinine (Ecrn), ergokryptinine (Ekrn), and ergocorninine (Econ). Considering that the main compounds and their epimers can be found together in contaminated samples, it is mandatory to evaluate all of them when an analytical method is applied to determine a possible contamination by EAs in food or feed [3,8].

Different methods have been reported for the analysis of EAs in different commodities (mainly cereals, feeds, and cereal-based foods), usually employing liquid chromatography (LC) with fluorescence or mass spectrometry (MS) detection modes [9]. However, in order to provide an unequivocal identification of major EAs and their epimers, LC or ultra-high performance LC (UHPLC) coupled with tandem mass spectrometry (MS/MS), usually applying electrospray ionization in the positive mode ESI(+), have become the techniques of choice [10,11,12,13]. In relation to the applied sample treatments, solid-liquid extraction followed by a clean-up step, if required, is usually used, or the so-called QuEChERS (quick, easy, cheap, effective, rugged, and safe) procedure, based on an extraction/partition step followed by a clean-up using dispersive solid-phase extraction (d-SPE). A mixture of acetonitrile (MeCN) and an alkaline solution, to avoid epimerization during the procedure, has been used as the extraction solvent for EAs [10,11,12]. All these aspects are summarized in recent reviews covering the advances of mycotoxin analysis, including EAs [14,15,16,17].

As stated before, most of the previous works are focused on the analysis of cereals, feed, and cereal-based products susceptible of contamination by EAs. Some of these products can be considered functional foods, due to their physiological benefits or protection against chronic diseases. In this sense, the benefits of oats are well-known: they are a source of fiber, vitamins, and antioxidants; they may reduce the risk of heart diseases, improve blood sugar control, help one to lose weight, and they are also used for skincare [18,19]. Some of these health claims have been authorized by the EFSA and the Food and Drug Administration (FDA) [20,21]. Oats are usually consumed for breakfast as flakes or oatmeal, but they can also be used as flour for baking. Moreover, food supplements (that is, products intended to provide a concentrated source of nutrients and other substances), including oat extracts or oat grass juices in their composition are available on the market. These products can also suffer from fungi and mycotoxin contamination, and this aspect must be a matter of concern, as highlighted in several research works and reviews [22,23,24,25]. The EFSA specifies that, regarding mycotoxin contamination, herbal products used in food supplements must comply with the current food legislation of the EU [26]. However, to the best of our knowledge, the presence of EAs in these kinds of cereal-derived products has not been studied to date.

Considering all of the above-mentioned aspects, in order to protect consumer health, the development of analytical methods for the determination of main EAs in cereal-based products (including food supplements) is of great importance. Therefore, in this work, a method based on a modified QuEChERS, combined with UHPLC–MS/MS, is proposed for the simultaneous extraction and quantification of the main EAs and their epimers in different oat-based products. The method was validated and applied to 25 different oat-based products destined for human consumption.

## 2. Results and Discussion

### 2.1. Optimization of the Sample Treatment

In this work, a QuEChERS procedure reported by Guo et al. for the analysis of EAs in cereals samples was modified [27] and applied for the analysis of bran, flakes, flour, tablets, and grass oat samples. Some important improvements, in terms of reduction of organic solvent consumption and a more efficient clean-up of the matrix, were achieved. Firstly, the volume of the extraction mixture (MeCN: 5 mM ammonium carbonate, 85:15, *v*/*v*) was reduced from 10 to 4 mL, which was enough to extract the studied EAs from 1 g of sample with satisfactory recoveries. In addition, to increase the sensitivity of the method, all of the supernatant volume was collected in all steps, in contrast with the standard QuEChERS procedure, in which only a part of the supernatant was considered.

**Figure 1:**
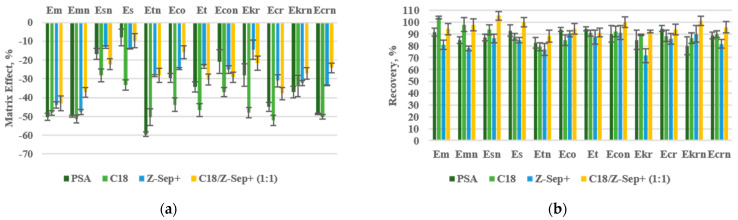
Optimization of the sorbent in the d-SPE step of the QuEChERS procedure. Effect of the kind of dispersive sorbent in the matrix effect (**a**); effect of the kind of sorbent in the recovery study (**b**).

Subsequently, to improve the extraction efficiency and to reduce the matrix effect (ME), different sorbents were tested in the d-SPE step, such as C18, Z-Sep+, primary-secondary amine (PSA), and a mixture of C18/Z-Sep+ (1:1). In all cases, an amount of 150 mg of sorbent was used. In general, the ME was considerably lower when Z-Sep+ was used (a sorbent recommended to remove lipids, fatty acids, and carbohydrates), but the recoveries were negatively affected in most cases in comparison with the other tested sorbents. On the other hand, when C18 or PSA were employed, better recoveries were obtained (the highest valueswere obtained with C18 in most cases, especially for Em and Emn), but the ME was also significantly higher for all the analytes. In view of these results, a mixture of C18 and Z-Sep+ (1:1) was tested. A compromise between satisfactory values for recoveries and ME of the studied analytes was achieved when this mixture was employed, as can be seen in Figure 1. Therefore, it was selected as a dispersive sorbent for further experiments. Afterwards, the amount of this mixture was investigated, using 90 and 150 mg. With 90 mg, the ME increased considerably in all cases without affecting the recovery results, meaning that a lower amount of dispersive sorbent was not enough for cleaning-up the extract, so 150 mg of C18:Z-Sep+ (1:1) was selected as optimum.

Finally, the extraction time was kept at 1 min to reduce the time of the sample treatment, also preventing epimerization. Moreover, high temperatures in the evaporation step were avoided, and the final extracts were dried at room temperature under a gentle stream of nitrogen.

### 2.2. Method Validation

For the validation of the analytical method, procedural calibration curves and parameters, such as limits of detection (LODs), limits of quantification (LOQs), ME, precision, and recoveries were studied using a blank oat flake sample as the representative matrix.

#### 2.2.1. Calibration Curves and Analytical Performance Characteristics

In order to compensate ME and the losses in the sample treatment, procedural calibration curves were established in the blank oat flake samples, spiked at six different concentration levels (2, 5, 10, 25, 50, and 100 µg/kg), processed in duplicate, and injected in triplicate. The peak area was considered as a function of the analyte concentration. LODs and LOQs were calculated as the minimum analyte concentrations with a signal-to-noise ratio equal of 3 and 10, respectively. The statistical parameters calculated by least-square regression, LODs and LOQs, as well as the root mean square error of calibration (RMSEC) and root mean square error of prediction (RMSEP) are shown in Table 1. The satisfactory determination coefficients (R^2^ > 99%) confirmed that the analytical responses for the studied EAs were linear over the studied ranges. Moreover, the low values for RMSEC and RMSEP indicated that the calibration model fits properly. Satisfactory LOQs (from 0.2 µg/kg for Emn to 3.2 for Em µg/kg) were obtained, being lower than those obtained with other QuEChERS–LC–MS/MS methods applied to the quantification of EAs in cereal samples [27,28]. 

#### 2.2.2. Precision

The precision of the proposed method was evaluated in terms of repeatability (intraday precision) and intermediate precision (interday precision). Repeatability was assessed in oat flake samples by application of the whole procedure to three samples (experimental replicates) spiked at two different concentration levels of each EA (5 and 50 μg/kg). All samples were analyzed on the same day and each extract was injected in triplicate (instrumental replicates). Intermediate precision was evaluated with a similar procedure, but analyzing one spiked sample in triplicate and per day, for three consecutive days. The results of the precision study, expressed as relative standard deviation (RSD, %) are shown in Table 2. In all cases, RSD values lower than 6.3% for repeatability and 15.0% for intermediate precision were obtained, in agreement with the recommendations for determination of the contaminants [29].

#### 2.2.3. Recovery Studies

Recovery experiments were carried out in blank oat flake samples previously analyzed to check the absence of detectable EAs. None of them gave a positive result above the LODs of the method. Recovery experiments were carried out in three samples spiked at two different concentration levels (5 and 50 μg/kg), and injected in triplicate. The results were compared with those obtained for extracts of blank samples submitted to the sample treatment and spiked with EAs just before the measurement. As summarized in Table 3, recoveries between 89.7 and 109% were obtained in all cases, fulfilling requirements of the performance criteria for quantitative methods of analysis [30].

#### 2.2.4. Evaluation of Matrix Effects

In order to evaluate the influence of ME on MS detection, cleaned-up extracts of blank samples were spiked with a solution mixture of EAs at two levels of concentration (5 and 50 μg/kg) and analyzed by UHPLC–MS/MS. Standard solutions of the EAs were prepared at the same levels of concentration and were also analyzed. The ME was calculated as 100× [(signal of spiked extract − signal of standard solution)/signal of standard solution]. A ME of 0% indicates the absence of the matrix effect, a ME below 0% involves signal suppression while a ME above 0% reveals signal enhancement. Table 3 shows the values of the ME and, as can be seen, signal suppression was not significant for most EAs, being lower than |20%|, except for Em, Emn (two levels), and Ecr and Ecrn (only for the lowest tested level). Anyway, procedural calibration was performed to compensate this ME.

### 2.3. Analysis of Oat-Based Products

**Figure 2:**
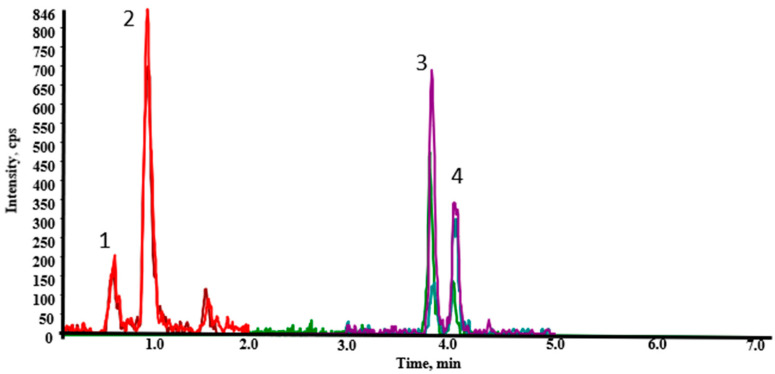
UHPLC-ESI (+)-MS/MS extracted ion chromatogram of naturally contaminated sample of oat bran with four EAs: (1) Em, (2) Emn, (3) Esn, (4) Es.

In order to check the applicability of the validated method, a total of 25 oat-based products—bran (11), flakes (5), hydroalcoholic extracts (2), juices (2), flours (2), tablets (2), and grass (1)—were analyzed to monitor the natural occurrence of EAs in this sort of products. For identification of EAs in the samples, the recommendations of the SANTE guideline were followed [30]. Thus, the samples, which met all these requirements and contained EAs at levels exceeding the LOQs would be considered positives. Only one sample of oat bran was positive for Em (7.2 μg/kg), Emn (1.1 μg/kg), Es (1.3 μg/kg), and Esn (1.1 μg/kg), showing, therefore, a total content of EAs of 10.7 μg/kg. A chromatogram of this naturally contaminated sample is shown in Figure 2.

## 3. Materials and Methods

### 3.1. Chemical and Reagents

All reagents were of analytical reagent grade and solvents were LC–MS grade. MeCN, methanol (MeOH), and ammonium carbonate were obtained from VWR (Barcelona, Spain). Formic acid eluent additive for LC–MS was supplied by Sigma Aldrich (Madrid, Spain). Z-Sep+ sorbent for clean-up was from Supelco (Bellefonte, PA, USA), while C18 and PSA sorbents were supplied by Agilent Technologies (Madrid, Spain).

Ultrapure water used throughout the work was obtained from a Milli-Q water purification system (18.2 MΩ cm, Milli-Q Plus system, Millipore Bedford, MA, USA).

### 3.2. Standards

Standards of Es, Eco, Ekr, Ecr, and the corresponding epimers, Esn, Econ, Ekrn, Ecrn, were purchased from Techno Spec (Barcelona, Spain), whereas Em, Et, Emn, and Etn were obtained from Romer Labs (Getzersdorf, Austria). Following the indications of the manufacturer, the standards were reconstituted in 5 mL of MeCN, to achieve concentrations of 500 μg/mL for the main EAs and of 125 μg/mL for the epimers. Immediately after this reconstitution, to avoid the rapid epimerization of EAs in the solution, intermediate dried stock solutions were prepared. For that, aliquots of individual or mixed standard solutions were placed into amber glass tubes, evaporated to dryness under a gentle stream of N_2_, and stored at −20 °C. These intermediate stock solutions were reconstituted in the required amount of MeCN just before use.

### 3.3. Instrumentation and Equipment

UHPLC–MS/MS experiments were performed in an Agilent 1290 Infinity LC (Agilent Technologies) coupled to an API 3200 triple quadrupole mass spectrometer (AB Sciex, Darmstadt, Germany) with electrospray ionization (ESI). The chromatographic separation was carried out using an Agilent Zorbax Eclipse Plus RRHD C18 column (50 × 2.1 mm, 1.8 µm). Analyst software (Version 1.6.3, AB Sciex) was used for acquisition and data analysis.

During the sample treatment procedure, a high-speed solids crusher (Hukoer, China), an evaporator system (System EVA-EC, from VLM GmbH, Bielefeld, Germany), a vortex-2 Genie (Scientific Industries, Bohemia, NY, USA), and a universal 320R centrifuge (Hettich Zentrifugen, Tuttlingen, Germany) were used.

### 3.4. Samples

A total of 25 oat-based samples, including some food supplements, were randomly purchased from different local markets in Granada (Spain). Oat-based products can be found in different presentations, so the following products were selected: oat bran (11), flakes (5), flours (2), hydroalcoholic extracts (2), juices (2), tablets (2), and grass (1). In order to obtain representative samples, several portions were taken from each unit, being thoroughly mixed. Samples were milled (when necessary) and/or homogenized and stored at room temperature prior to the extraction step.

Method optimization and validation were performed using a blank oat flake sample as the representative matrix, which was previously analyzed to ensure the absence of EAs.

### 3.5. Sample Preparation

Briefly, a portion of 1.0 g of the homogenized sample (bran, flakes, flour, tablets, and grass) was placed into a 50-mL falcon tube with a conical bottom, and then 4 mL of MeCN and 5 mM ammonium carbonate (85:15, *v*/*v*) were added. The mixture was shaken by vortex for 1 min, and afterwards, the sample was centrifuged at 9000 rpm for 5 min at 4 °C. Subsequently, the whole upper layer was collected and placed into a 15-mL falcon tube containing 150 mg of a mixture of C18:Z-Sep+ (1:1) as dispersive sorbent for the clean-up step. Then, the 15-mL tube was vigorously shaken for 1 min and centrifuged at 8784 g for 5 min at 4 °C. Finally, the upper layer was fully transferred to a glass tube and evaporated to dryness under a gentle stream of N_2_. The residue was reconstituted with 750 µL of a mixture of MeOH:water (50:50, *v*/*v*) and passed through a 0.22 µm nylon membrane filter before injection into the UHPLC–MS/MS system.

“Direct-injection” and “dilute-and-shoot” methods were used in the case of hydroalcoholic extract samples and juice samples, respectively, as no extraction or clean-up steps were required.

### 3.6. UHPLC–MS/MS Conditions

The chromatographic separation of EAs was carried out using a C18 Zorbax Eclipse Plus RRHD column (50 × 2.1 mm, 1.8 µm). The mobile phase consisted of 0.3% formic acid aqueous solution (solvent A) and MeOH with 0.3% formic acid (solvent B) at a flow rate of 0.4 mL/min. The eluent gradient profile was as follows: 0–6 min 30–60% B; 6–9 min 60% B; 9–10 min 60–30% B; 10–12 min 30% B. The column temperature was set at 35 °C and the injection volume was 5 μL. In order to minimize epimerization, the injection sample sequence was limited to 12 h. Moreover, control standard solutions of EAs were injected at the beginning, middle, and end of each analysis sequence.

The mass spectrometer operated in the positive electrospray ionization (ESI+) mode under the multiple reaction monitoring (MRM) conditions, shown in Table S1. MS parameters for the analysis were established as follows: temperature of the source 500 °C; collision gas (nitrogen) 5 psi; voltage of the ion spray 5 kV; curtain gas (nitrogen) 30 psi; nebulizing gas (GAS 1), and drying gas (GAS 2), in both of them, nitrogen was set at 50 psi. In all cases, a precursor ion and two product ions were studied. The monitored ions were the protonated molecules [M + H]^+^, except for Esn, Etn, Econ, Ecrn, and Ekrn, where the signal at *m/z* corresponding to [M − H_2_O + H]^+^ was higher than that of the protonated molecules, in accordance with a previous work [10].

**Figure 3:**
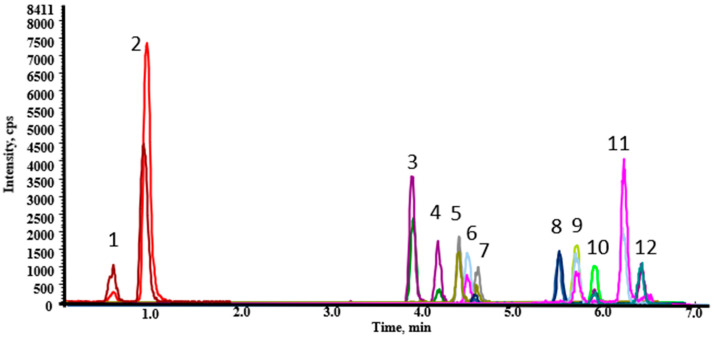
UHPLC-ESI (+)-MS/MS extracted ion chromatogram of a blank oat sample spiked with EAs at 10 µg/kg: (1) Em, (2) Emn, (3) Esn, (4) Es, (5) Etn, (6) Eco, (7) Et, (8) Econ, (9) Ekr, (10) Ecr, (11) Ekrn, (12) Ecrn.

Under optimum conditions, EAs and their epimers were separated and detected in less than 7 min (Figure 3). EAs were identified by retention times and mass spectra as shown in Figure S1.

## 4. Conclusions

In this work, an effective and sensitive QuEChERS–UHPLC–MS/MS method, which enabled the quantification of the six major EAs, as well as their corresponding epimers, was validated and applied to the analysis of a variety of oat-based samples. The modifications carried out in the standard QuEChERS procedure improved the sensitivity and the effectiveness of the method. On the one hand, the reduction of the extraction solvent volume resulted in an increase in the sensitivity, since the LOQs obtained (below 3.2 μg/kg) were significantly lower when compared with those obtained with similar procedures for EAs determination in cereal samples. On the other hand, using a mixture of C18/Z-Sep+ (1:1) as dispersive sorbent, the ME was reduced, being below |20%| for most analytes studied in such complex matrixes. Moreover, analyzed samples were representative of the wide range of presentations in the market of oat-products. To the best of our knowledge, this is the first time that EAs have been explored in such a variety of oat-based functional foods, including food supplements. Although only one sample of oat bran was contaminated with EAs, it shows that despite the improvements in industrial grain processing, contamination by EAs must be considered, especially in cereal-based processed foods.

## Figures and Tables

**Table 1:** Statistical and performance characteristics of the proposed method for the determination of main EAs and their epimers in oat flake samples.

Analyte	Linear Regression Equation	Linear Range (µg/kg)	Linearity (R^2^, %)	LOD (µg/kg)	LOQ (µg/kg)	RMSEC (µg/kg)	RMSEP (µg/kg)
Em	y = 152.0x − 112.6	3.2–100	99.5	1.0	3.2	2.5	3.6
Emn	y = 4971.3x − 1979.2	0.2–100	99.6	0.1	0.2	2.1	3.6
Es	y = 1590.4x − 355.9	1.0–100	99.6	0.5	1.0	2.1	2.4
Esn	y = 941.7x + 506.3	0.9–100	99.4	0.6	0.9	2.7	2.9
Et	y = 565.3x − 1045.3	2.0–100	99.5	0.6	2.0	2.5	3.2
Etn	y = 543.5x + 399.4	1.7–100	99.6	0.5	1.7	2.0	3.1
Eco	y = 867.4x + 17.45	1.4–100	99.8	0.4	1.4	1.6	2.9
Econ	y = 692.9x + 373.2	1.4–100	99.8	0.4	1.4	1.6	2.5
Ekr	y = 935.9x − 1148.7	1.5–100	99.6	0.5	1.5	2.3	2.0
Ekrn	y = 935.9x − 1148.7	1.9–100	99.6	0.6	1.9	3.2	2.6
Ecr	y = 546.0x − 223.4	1.9–100	99.6	0.6	1.9	2.0	2.4
Ecrn	y = 677.3x − 304.9	1.6–100	99.4	0.5	1.6	2.6	3.5

**Table 2:** Precision of the proposed method for the determination of main EAs and their epimers in spiked oat flake samples.

	Repeatability, % RSD (n = 9)		Intermediate Precision, %RSD (n = 9)	
	5 µg/kg	50 µg/kg	5 µg/kg	50 µg/kg
Em	5.0	3.2	15.0	10.9
Emn	4.2	2.1	13.6	9.8
Es	3.7	3.2	7.9	6.9
Esn	5.7	3.2	14.3	10.8
Et	6.2	3.8	15.0	11.7
Etn	5.5	4.8	14.0	8.2
Eco	6.3	3.2	12.0	10.4
Econ	4.6	2.2	10.2	7.0
Ekr	5.4	3.7	12.8	7.3
Ekrn	4.2	3.7	14.7	9.4
Ecr	6.2	4.0	11.3	6.7
Ecrn	5.1	4.6	14.3	7.4

**Table 3:** Matrix effect and recovery studies of the proposed UHPLC–MS/MS method for the determination of EAs and their epimers in spiked oat flake samples.

	Matrix Effect (%) (n = 9)		Recovery (%) (n = 9)	
	5 µg/kg	50 µg/kg	5 µg/kg	50 µg/kg
Em	−40.4	−39.1	92.4	101
Emn	−36.6	−28.2	90.5	97.1
Es	−8.8	−7.2	105	102
Esn	−8.6	−8.1	97.2	102
Et	−15.5	−10.7	89.7	91.6
Etn	−9.3	−6.2	109	97.9
Eco	−9.6	−5.3	109	90.7
Econ	−13.4	−9.0	105	98.3
Ekr	−14.0	−8.5	106	91.6
Ekrn	−19.7	−11.7	93.1	98.7
Ecr	−22.2	−18.7	90.0	92.1
Ecrn	−24.1	−18.6	103	106

## Data Availability

Not available.

## Supplementary Materials

The following are available online, Table S1: MS parameters for the different target analytes studied in the proposed UHPLC–MS/MS method. Figure S1. Identification of 12 EAs.

## Sample Availability

Samples of the compounds are available from the authors.
