# Supplementary material for: Determination of the Main Ergot Alkaloids and Their Epimers in Oat-Based Functional Foods by Ultra-High Performance Liquid Chromatography Tandem Mass Spectrometry
Source: Molecules. 2021 Jun 18;26(12):3717. doi: 10.3390/molecules26123717 (PMC8234484; doi:10.3390/molecules26123717)

SUPPLEMENTARY MATERIAL

DETERMINATION OF THE MAIN ERGOT ALKALOIDS AND THEIR EPIMERS IN OAT-BASED FUNCTIONAL FOODS BY ULTRA-HIGH PERFORMANCE LIQUID CHROMATOGRAPHY TANDEM MASS SPECTROMETRY

Laura Carbonell-Rozas, Laura Gámiz-Gracia, Francisco J. Lara, Ana M. García-Campaña*.

Department of Analytical Chemistry, Faculty of Sciences, University of Granada

Avda. Fuente Nueva s/n, 18071, Granada, Spain

*Corresponding author: amgarcia@ugr.es

Table S1. MS parameters for the different target analytes studied in the proposed UHPLC-MS/MS method.

	Precursor ion (m/z)	Molecular ion	DP[a]	EP[a]	Product ions[b]	CE[a]	CXP[a]
**Em**	326.0	$[M+H]^+$	51	5.0	223.0 (Q)	23	4.0
					208.1 (I)	37	4.0
**Emn**	326.0	$[M+H]^+$	46	6.0	208.1 (Q)	39	6.0
					223.1 (I)	33	6.0
**Es**	548.2	$[M+H]^+$	61	5.0	208.2 (Q)	57	4.0
					223.1 (I)	45	8.0
**Esn**	530.2	$[M-H_2O+H]^+$	66	6.5	223.2 (Q)	37	6.0
					263.1 (I)	33	6.0
**Et**	582.2	$[M+H]^+$	56	7.0	208.2 (Q)	55	4.0
					223.2 (I)	45	4.0
**Etn**	564.2	$[M-H_2O+H]^+$	66	6.0	223.0 (Q)	41	6.0
					297.1 (I)	33	6.0
**Eco**	562.2	$[M+H]^+$	46	4.5	268.1 (Q)	33	6.0
					208.2 (I)	55	4.0
**Econ**	544.2	$[M-H_2O+H]^+$	61	8.5	277.1 (Q)	31	6.0
					223.1 (I)	37	6.0
**Ekr**	576.2	$[M+H]^+$	86	6.0	208.3 (Q)	59	6.0
					268.1 (I)	31	6.0
**Ekrn**	576.2	$[M-H_2O+H]^+$	36	7.0	223.0 (Q)	45	6.0
					558.0 (I)	21	6.0
**Ecr**	610.2	$[M+H]^+$	56	6.5	268.2 (Q)	35	6.0
					208.1 (I)	57	6.0
**Ecrn**	592.2	$[M-H_2O+H]^+$	71	7.5	305.1 (Q)	33	4.0
					223.2 (I)	39	6.0

[a] Declustering potential (DP), Entrance potential (EP), Collision Cell Exit Potential (CXP) and Collision Energy (CE). All expressed in voltage.
[b] Product ions: (Q) Transition used for quantification, (I) Transition employed to confirm the identification.

Figure S1. Identification of 12 EAs.

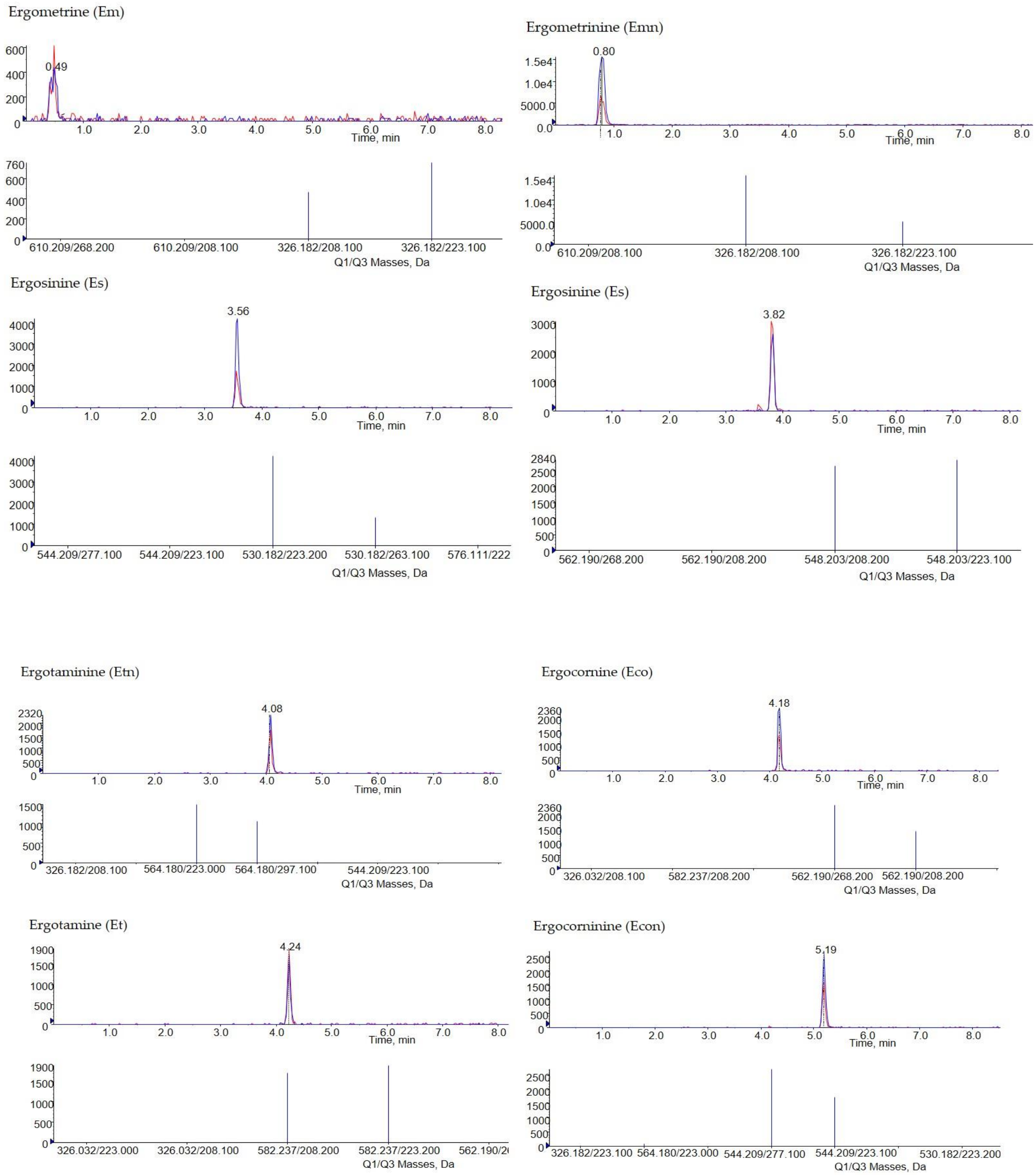

Figure S1. Identification of 12 EAs.

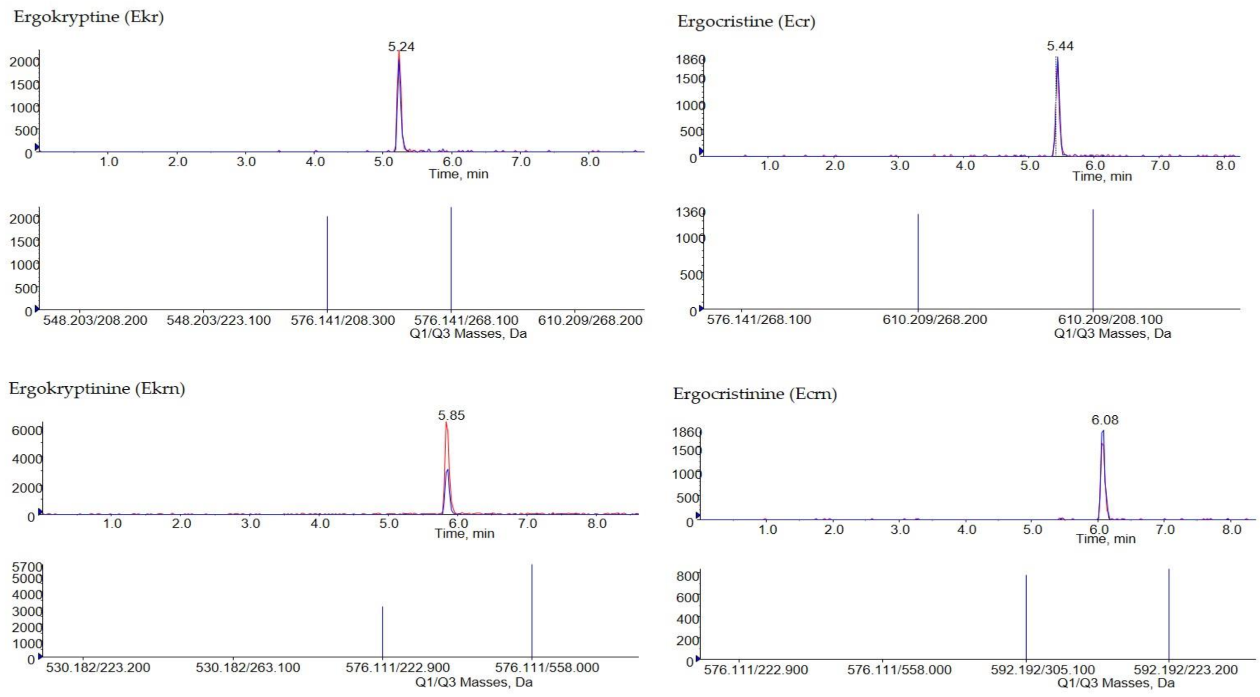

Supplement: Supplementary file 1 [file molecules-26-03717-s001.zip › molecules-1253518-supplementary.pdf]
